# The Safety of Ingested Caffeine: A Comprehensive Review

**DOI:** 10.3389/fpsyt.2017.00080

**Published:** 2017-05-26

**Authors:** Jennifer L. Temple, Christophe Bernard, Steven E. Lipshultz, Jason D. Czachor, Joslyn A. Westphal, Miriam A. Mestre

**Affiliations:** ^1^Department of Exercise and Nutrition Sciences, University at Buffalo, Buffalo, NY, USA; ^2^Department of Community Health and Health Behavior, University at Buffalo, Buffalo, NY, USA; ^3^Aix Marseille Univ, INSERM, INS, Inst Neurosci Syst, Marseille, France; ^4^Wayne State University School of Medicine, Children’s Hospital of Michigan, Detroit, MI, USA

**Keywords:** caffeine, energy drinks, pregnancy, children, adolescence

## Abstract

Caffeine is the most widely consumed psychoactive drug in the world. Natural sources of caffeine include coffee, tea, and chocolate. Synthetic caffeine is also added to products to promote arousal, alertness, energy, and elevated mood. Over the past decade, the introduction of new caffeine-containing food products, as well as changes in consumption patterns of the more traditional sources of caffeine, has increased scrutiny by health authorities and regulatory bodies about the overall consumption of caffeine and its potential cumulative effects on behavior and physiology. Of particular concern is the rate of caffeine intake among populations potentially vulnerable to the negative effects of caffeine consumption: pregnant and lactating women, children and adolescents, young adults, and people with underlying heart or other health conditions, such as mental illness. Here, we review the research into the safety and safe doses of ingested caffeine in healthy and in vulnerable populations. We report that, for healthy adults, caffeine consumption is relatively safe, but that for some vulnerable populations, caffeine consumption could be harmful, including impairments in cardiovascular function, sleep, and substance use. We also identified several gaps in the literature on which we based recommendations for the future of caffeine research.

## Introduction

Caffeine is the most widely consumed psychoactive drug in the world (1) and one of the most comprehensively studied ingredients in the food supply. It occurs naturally in the leaves and seeds of many plants and has a taste bitter enough to deter pests (2). Natural sources of dietary caffeine include coffee, tea, and chocolate. Synthetic caffeine is also added to products to enhance their stimulant properties. Historically, this addition was limited to soda-type beverages, but over the past decade, caffeine has been added to a diverse variety of foods and non-food items to promote arousal, alertness, energy, and elevated mood (3–5). This recent increase in caffeine-containing food products, as well as changes in patterns of consumption of the more traditional sources of caffeine, has increased scrutiny by health authorities and regulatory bodies of the overall consumption of caffeine and its potential cumulative effects on behavior and physiology. Of particular concern is the rate of caffeine intake among populations potentially vulnerable to its negative effects. Health and regulatory authorities have recently highlighted the risk of consumption among pregnant and lactating women, children, adolescents, young adults, and people with underlying heart and other health conditions.

In light of these concerns, we conducted a comprehensive review of all relevant published clinical and intervention trials, observational studies, systematic reviews, meta-analyses, and expert reviews on the use and safety of caffeine in humans, complemented where needed (e.g., for aspects of safety or mechanisms of action) with evidence from animal studies. We evaluated the strengths and limitations of the evidence on the safety of ingested caffeine, specifically focusing on the safety of caffeine-containing foods (e.g., beverages and solid foods). We summarize here what is known and what remains to be learned about caffeine intake and safety in healthy and vulnerable populations and highlight needed research.

## Dietary Sources of Caffeine

Adults commonly consume caffeine in coffee and tea, both of which contain natural caffeine in their leaves or beans (6). Energy drinks often contain caffeine from natural products such as extracts from guarana leaves. In addition to coffee, tea, and energy drinks, caffeine is also naturally present in cocoa beans and thus in chocolate. The amount of caffeine in chocolate varies by the percentage of cocoa it contains, with 100% cocoa chocolate (unsweetened baking chocolate) containing around 240 mg caffeine/100 g, 55% cocoa (bittersweet) containing 124 mg caffeine/100 g, and 33% cocoa (milk chocolate) containing 45 mg caffeine/100 g (7). Synthetic caffeine is also added to soda and energy drinks (8), which are commonly consumed by children and adolescents worldwide, and to other food and non-food products with niche markets for subsets of consumers, such as juice, chewing gum, water, cookies, hot sauce, candy, beef jerky, mints, syrup, waffles, shampoo, soap, lip balm, eye cream, body scrub, and body lotion. These products are primarily marketed with claims that they provide energy, alertness, or are “age-defying.” Last year, the FDA announced that it will begin investigating the safety of caffeine added to food products, with a special emphasis on children and adolescents.^1^

Caffeine is a constituent of many over-the-counter pain relievers and prescription drugs because the vasoconstricting and anti-inflammatory effects of caffeine act as a compliment to analgesics, in some cases increasing the effectiveness of pain relievers by up to 40% (9–14). Caffeine is used for general pain relief in medications such as Midol™ and Vanquis™, which contain doses ranging from 33 to 60 mg. It is used therapeutically in combination with ergotamine to treat migraine headaches and in combination with non-steroidal anti-inflammatory analgesics. Anacin™, Excedrin™, Goody’s™ headache powder, and pain reliever plus contain between 32 and 65 mg of caffeine, and prescription headache medications, including Fiorinal, Orphenadrine, and Synalgos, contain between 30 and 60 mg of caffeine.

Alone, caffeine is used as a somnolytic to counteract drowsiness (e.g., NoDoze™ and Vivarin™ each contain 200 mg of caffeine), to enhance seizure duration in electroconvulsive therapy, and to treat respiratory depression in neonates, postprandial hypotension, and obesity (15–18). Similar synergistic additive effects of caffeine and medications also occur in treatments for asthma and gall bladder disease, attention deficit-hyperactivity disorder, shortness-of-breath in newborns, low blood pressure, and weight loss (19–24). Between 50 and 200 mg of caffeine is added to some weight-loss supplements (Dexatrim™, Hydroxycut™, and Nutrisystem™ Energi-Zing Shake) for its purported effects on appetite suppression and increased metabolism (25).

## Estimates of Caffeine Consumption

Recent estimates in adults suggest that more than 85% of adults in the U.S. regularly consume caffeine, with an average daily intake of about 180 mg/day, about the amount of caffeine in up to two cups of coffee (6, 26). Among children and adolescents, caffeine use appears to be either stable or slightly decreasing over time, despite the influx of new caffeine-containing products on the market. For example, a study by Ahluwalia and Herrick using NHANES data reports that about 75% of U.S. children between 6 and 19 years old consume caffeine, with an average consumption of 25 mg/day in children 2–11 years old and 50 mg/day in children 12–17 years old (8). Another study also using the NHANES dataset reports average caffeine consumption in children and adolescents as 35 mg/day, with 4–8 years old consuming 15 mg/day, 9–13 years old consuming 26 mg/day, and 14–19 years old consuming 61 mg/day (27).

Coffee consumption varies worldwide: Finland and Norway are at the top of the list, with averages of 9.6 and 7.2 kg of coffee consumed per capita per year. The U.S. ranks 22nd, with 3.1 kg. A 1984 study showed that Canada and the U.S. had per capita rates of caffeine consumption that were triple the worldwide average but that were still half of what was consumed in countries such as Sweden and the United Kingdom (U.K.) (28). A more recent study from the Canadian Community Health Survey found that coffee was the second most popular drink among Canadian adults, with water being the first (29). The U.K.’s National Diet and Nutrition Survey also collected information on caffeine consumption through foods and beverages from adults and children. These data show that, on average, adults in the U.K. consume about 130 mg/day of caffeine and that children consume about 35 mg/day (30). A study from Japan using 4-day food diaries reported average daily caffeine consumption as about 260 mg/day in adults (31). Finally, people in Finland, Norway, the Netherlands, and Sweden are consistently reported to drink the most caffeine, primarily from coffee. However, these estimates are derived from sales of coffee and not from surveys of individual intake.

## Trends in Caffeine Consumption

Trends in caffeine consumption have been stable among adults for the past two decades (6). Among children aged 2–19 years old, caffeine consumption increased significantly from the 1970s through the 1990s (5, 32). This increase was also marked by a decrease in dairy consumption and an increase in soda consumption (32). More recent data suggest that caffeine consumption has remained stable among this age group since the 1990s (8, 33), a finding similar to that in adults. This stability is somewhat surprising, given the marked increase in the number, variety, and availability of caffeinated beverages introduced in the past decade. Some researchers speculate that this stability reflects a lag in data collection or in consumption trends from when products are introduced to the market to when data are collected (for example, the most recent NHANES data on caffeine consumption are from 2011). Another potential explanation is that a possible decline in consumption among younger children has been offset by increased consumption among older adolescents and young adults attracted to the increasing number of new caffeine-containing products. Targeted marketing strategies seem to support this explanation. Advertisements for caffeinated energy drinks, the fastest growing segment of the beverage market (34, 35), are specifically aimed at adolescent and young adult males (36, 37). Given the popularity and prevalence of energy drinks, caffeine consumption could reasonably be expected to increase quickly among children and adolescents.

Caffeine intake usually begins in childhood, most often in the form of chocolate, soda, and chocolate milk (8). As children become adolescents, they increase consumption of soda and begin to add beverages with greater caffeine content, such as coffee and energy drinks (8). Average caffeine intakes increase from about 50 mg/day in childhood (aged 2–11 years) to 180 mg/day in adulthood (6). This amount is about 2 mg/kg/day in children, 2.4 mg/kg/day in women, and 2.0 mg/kg/day in men. This shift in absolute caffeine intake from childhood to adulthood is related to changes in the pattern of consumption, with adults adopting a more regular, daily pattern of consumption relative to children (6). In addition, the dietary sources of caffeine shift over the lifespan: adults primarily consume coffee and tea, whereas children and adolescents consume primarily soda and chocolate, which contain much lower amounts of caffeine.

The pattern of caffeine use changes across the lifespan has not been studied, but tolerance to the effects of caffeine has been speculated to increase the desire for larger doses to reverse the impact of overnight caffeine withdrawal (38). In addition, once caffeine intake is great enough to disrupt sleep, or if sleep duration is shortened by other factors, caffeine is often used to promote morning arousal, which can further disrupt sleep, creating a pattern in which caffeine is both the cause and the cure for too little sleep (38, 39). Variations in caffeine sensitivity and consumption may relate to polymorphisms in enzymes that degrade caffeine and in adenosine receptors, which are the primary targets of caffeine (40).

## The Pharmacokinetics of Caffeine

Caffeine works by binding to adenosine receptors located in the central and peripheral nervous systems as well as in various organs, such as the heart, and blood vessels. Adenosine is a molecule involved in numerous biochemical pathways, mostly for energy transfer (in the form of adenosine triphosphate, the basic fuel of cells) and signaling. Adenosine is a neuromodulator that can promote sleep, affect memory and learning, and protect cells after insults. Adenosine can also act on several types of cognate receptors: for example, A1, A2a, A2b, and A3, which are G-coupled proteins. In the central nervous system, activating A1 receptors inhibits the release of neurotransmitters, whereas activating A2a receptors promotes their release (41). During early stages of brain development, the predominant effect of caffeine is to antagonize type 2A adenosine receptors, slowing down the migration speed of some neurons (42). At toxic doses (i.e., extreme doses that humans rarely absorb), caffeine can alter other cellular functions, releasing Ca^2+^ from intracellular stores at lethal levels (43). The toxic dose effects are not considered here because, although they are of great concern to the medical profession and may be on the rise, they are still rare compared to other, non-lethal caffeine effects and the precise mechanism of caffeine toxicity has not been investigated in humans.

### Absorption and Metabolism

Caffeine is usually ingested. Caffeine is soluble in water and lipids, easily crosses the blood–brain barrier, and can be found in all body fluids, including saliva and cerebrospinal fluid. Importantly, caffeine ingested by women perinatally will be present in the umbilical cord and breast milk. Hence, it will also be present in the fetus and in breastfed infants. Caffeine is absorbed rapidly and totally in the small intestine in less than 1 h (44) and diffuses rapidly in other tissues (45). Absorption by the small intestine does not seem to vary by sex, genetic background, environmental factors, or other variables (46), although specific studies are still needed to confirm this premise. Caffeine concentrations peak in saliva 45 min after ingestion (47) and in serum after about 2 h (48). Caffeine has a relatively long half-life of 3–7 h in adults. In neonates, the half-life is even longer—between 65 and 130 h—because of their immature kidneys and liver. Peak concentrations are important because the effects of caffeine depend in part on the length of time it remains in tissues. Clearly, the effects are age dependent and depend on complex genetic and environmental interactions.

Caffeine is primarily metabolized in the liver by the cytochrome P450 oxidase enzyme system; in particular, by the CYP1A2 enzyme. However, this oxidase enzyme system is also present in other tissues, including the brain (49). Caffeine metabolism is affected by several factors, described in detail below.

### Genetic Variation

The *CYP1A2* gene, which encodes for a cytochrome P450 enzyme, has a large genetic variability. At least 150 single-nucleotide polymorphisms can accelerate caffeine clearance (50). The metabolic consequences of this polymorphism on caffeine downstream effects should be studied in humans. Genetic variation (i.e., increased or decreased activity of the cytochrome P450 oxidase enzyme) may increase or decrease the possible harmful effects of caffeine (e.g., during pregnancy) and any beneficial effects (e.g., on memory and learning during aging or in pathologies, such as Alzheimer’s disease). The half-life of caffeine may also be increased in liver diseases, which decreases P450 activity (50).

The molecular targets of caffeine, namely the adenosine receptors, also have great genetic variability. For example, common variants of the gene encoding for the A2a receptor can disrupt sleep (51) or cause anxiety in some individuals (52) after ingesting caffeine. More studies are needed to determine the effects of genetic variants on the consequences of caffeine consumption (53), not only in the central nervous system but also in other organs, such as the heart (40).

### Circadian Rhythms

The expression of the cytochrome P450 epoxygenases is regulated in a circadian manner (54). Although this effect was discovered in cultured rodent cells, it may apply to many species, including humans (55). The implications are particularly important because the effects of caffeine (at least the duration of its activity) will differ during the circadian cycle. Because caffeine can alter sleep, it may also change the circadian rhythm, leading to a change in expression patterns for the cytochrome P450. One interesting hypothesis is whether caffeine consumption in adolescents and adults disrupts the expression of P450 in relation to its circadian rhythm. If the expression is downregulated, the effects of caffeine could be prolonged and produce a negative feedback loop.

### Steroid Hormones

The cytochrome P450 oxidase enzyme system is the same enzyme that metabolizes steroid hormones (56). Thus, steroid hormones slow caffeine metabolism. In women, this effect slows the metabolism of caffeine during pregnancy and when taking oral contraceptives (57). However, studies have not found marked differences in caffeine metabolism between the luteal and follicular phases of the menstrual cycle (57, 58). Oral contraceptives tend to double the half-life of caffeine (59).

### Pregnancy

The half-life of caffeine is on average 8.3 h longer during pregnancy and may be as much as 16 h longer than usual (60, 61). This longer half-life means that the effects of caffeine will be longer lasting in women and in the fetus. Given the effects that caffeine may have on brain development, this increased half-life in pregnant women should be taken into account when considering safety issues.

### Infancy

Caffeine is eliminated more slowly during early infancy, requiring perhaps 80 h in preterm and healthy-term neonates, because of the reduced efficiency of cytochrome P450 (62, 63). Elimination is likely to be at least as slow in the fetus. Fetal exposure to caffeine during pregnancy may potentially have long-lasting effects, especially in the brain. By age 6 months, infants eliminate caffeine at the same rate as that of adults (62).

### Substance Use

Cigarette smoking doubles the rate of caffeine clearance by increasing liver enzyme activity, which may explain the higher rate of caffeine consumption among smokers (64). Substantial alcohol intake increases the half-life of caffeine and decreases its clearance (65).

## Central and Peripheral Effects of Caffeine

The general effects of caffeine on body functions are summarized in Table 1.

**Table 1:** **Summary of outcome measures investigated**.

Outcome	Impact of caffeine	Comments
Cognitive effects	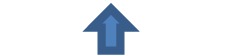	More effective in withdrawn and fatigued individuals
Neurological disorders	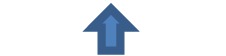	More pronounced benefits in women
Pain relief	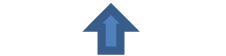	Works along with other pain relievers to improve their effectiveness
Cardiovascular function	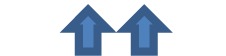	Dose-dependent effects on BP and HR. Harmful in cardiac patients
Vascular system	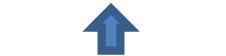	Caffeine causes vasoconstriction. Can increase risk for myocardial ischemia
Reproductive effects	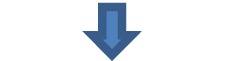	High caffeine increases risk of miscarriage
Congenital anomalies	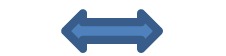	No clear association with caffeine
Birth weight	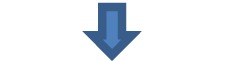	Negative correlation between caffeine intake and birth weight
Lactation	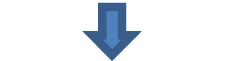	Could increase fussiness and impair sleep in infants
Behavioral disorders in children	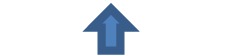	Energy drink consumption is positively associated with negative behavioral outcomes
Sleep disturbance	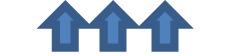	Caffeine disrupts sleep in all populations studied
Death	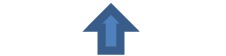	Rare
Cancer	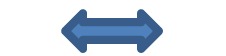	No clear association, but few studies
Unstable bladder	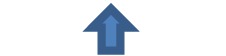	Primarily in women with preexisting bladder symptoms
Drug Interactions	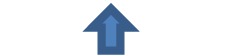	Potential negative interactions with many medications
Hydration and diuresis	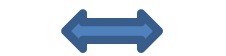	No clear relationship

*Arrows indicate whether caffeine increases, decreases, or has no impact on the outcome and the number of arrows indicates the strength of the relationship*.

### Cognitive Effects

Caffeine can influence objective and perceived cognitive performance by increasing alertness and wakefulness (66–68). Acute caffeine can also improve performance on memory tasks (69, 70). Finally, caffeine improves psychomotor vigilance, such as reaction time (71–73). The impact of caffeine appears to be greater under conditions that would negatively impact performance, such as acute caffeine withdrawal (74–76) or sleep deprivation (71, 77). In fact, studies that have employed long-term caffeine withdrawal methodology have consistently failed to find cognitive enhancing effects of acute caffeine (78–82). Nevertheless, in 2001, the Institute of Medicine’s Food and Nutrition Board Committee on Military Nutrition Research reported that ingesting 150 mg of caffeine enhances cognitive performance for at least 10 h (83), and this recommendation has not been updated in light of more recent empirical findings.

Numerous preclinical studies have found that antagonizing adenosine receptors, including with caffeine, has neuroprotective effects during aging and in neurological disorders by slowing cognitive decline and the progression of the disorders [reviewed in Ref. (84, 85)]. Based on these animal studies, several large longitudinal clinical studies in different countries have established an inverse relationship between coffee consumption and memory decline during normal aging (86–88). However, a study of 4,200 women and 1,800 men reported that caffeine consumption reduced cognitive decline only in women (69). In addition, a more recent study in a small group of women (89) failed to replicate the findings of the Ritchie study, demonstrating that more work is needed to understand the relationship between habitual caffeine consumption and cognitive performance. Finally, large cohort studies of men and women have also found an inverse relationship between caffeine consumption and the risk of Parkinson’s disease (90–92) and Alzheimer’s disease (93–95).

### Pain Relief

Caffeine has long been used to treat pain. However, its pain-reduction effects were not properly studied until 1984, when Lachance (96) documented that additive caffeine reduced the dose of acetaminophen necessary to achieve the target of a 40% reduction in pain scores (96). Since then, the vasoconstricting action of caffeine, secondary to adenosine receptor antagonism, has been associated with pain relief (97). Several studies have reported that acute dietary caffeine consumption can reduce pain (98, 99). In addition, caffeine in doses of between 300 and 500 mg can soothe post-dural puncture headaches, which is the most common complication of lumbar puncture procedures (100).

### Cardiovascular Effects

In general, acute intake of caffeine stimulates a modest increase in blood pressure (both systolic and diastolic), effects on heart rate (bradycardia or tachycardia, depending on dose), and neuroendocrine effects (release of epinephrine, norepinephrine, and renin) (101). These effects suggest that the mechanism of action is an increase in intracellular calcium concentrations, the release of norepinephrine, and the sensitization of dopamine receptors. These events may lead to supraventricular and ventricular tachyarrhythmias, especially at high doses. One proposed mechanism for caffeine-related cardiac arrhythmias is, again, the blockade of adenosine receptors (102, 103).

Patients with cardiac disease are often warned about the potential harmful effects of caffeine. For example, 94% of several hundred physicians from Minnesota and Vermont recommended reducing or stopping caffeine for patients reporting heart palpitations (104). However, this advice has been based primarily on anecdote and folklore (105, 106). Many of caffeine’s health effects occur after sympathetic excitation. Today, however, data suggest that caffeine does have cardiac effects, and arrhythmia is among them (107). Moreover, effects that do exist differ by dose and between habitual and non-habitual users. This severity of these threats often depends on such factors as preexisting medical conditions as well as the quantity of the ingredients taken and the length of time a person has been exposed to these substances. Many of the ingredients that include caffeine alone or in combination with other active substances have the potential to interact with prescription and over-the-counter medications. At typical caffeine doses, however, studies have documented mild changes in heart rate and blood pressure, a slight increase in sympathetic activity, and small changes in cardiac electrophysiological properties (105, 108–110).

### Vascular System Effects

Caffeine is believed to improve endothelial cell function at rest by increasing intracellular calcium concentrations, which stimulates the expression of endothelial nitric oxide synthase, which in turn stimulates the endothelial cells to produce nitric oxide. The nitric oxide then diffuses into vascular smooth muscle, which lies just underneath the endothelial cells, causing vasodilation (111). Caffeine can also bind directly to the vascular smooth muscle cell receptors and, through similar mechanisms, cause vasoconstriction (112).

The above information not withstanding, consuming caffeine immediately before or during exercise can be harmful and may increase the risk for myocardial ischemia (113). Indirect laboratory measures indicate that caffeine consumed immediately before exercising substantially reduces myocardial blood flow in healthy individuals (114). Several mechanisms may explain this reduction (114), including the ability of caffeine to block adenosine receptors that modulate coronary vasomotor tone. This vasoconstrictive effect might be more pronounced among caffeine-naïve individuals or in those who quickly ingest higher amounts of caffeine: for example, by consuming energy drinks. When caffeine blocks adenosine receptors, it reduces the ability of the coronary arteries to improve their flow commensurate with the increased myocardial demand of exercise, which could result in supply demand ischemia (114).

## Caffeine Toxicity

Seifert et al. (115) examined data from calls to the U.S. National Poison Data System made between October 1, 2010 and September 30, 2011 related to caffeine exposure and energy drink consumption (115). Of 2.3 million calls, 4,854 (0.2%) were energy drink related. Of the 1,480 calls related to exposures not involving alcohol, 51% concerned children under the age of 6, and 77% were the result of unintentional ingestion. The overall incidence of moderate-to-major adverse effects of energy drink-related toxicity was 15.2% for non-alcoholic energy drinks. The seven cases with major adverse effects consisted of three with seizure, two with non-ventricular dysrhythmia, one with ventricular dysrhythmia, and one with tachypnea. Of the same 1,480 calls, 946 concerned products containing caffeine only and 534 concerned products with caffeine-containing additives, such as guarana (a plant whose seeds are high in caffeine) or taurine (a naturally occurring organic acid often used as a nutritional supplement). Compared to energy drinks with additives, caffeine-only exposures involved a significantly greater proportion of cases less than 6 years old (50.7%) and a greater proportion of unintentional exposures (76.7%). The proportion of cases involving additives referred to a health-care facility was also significantly greater, as was the incidence of toxic effects of any severity. One caveat to this study is that information on preexisting medical conditions was not available for the cases studied. Research in this area should attempt to include and account for preexisting health conditions.

Researchers have also expressed concern about unintentional caffeine consumption and an increase in overconsumption of caffeinated energy drinks in children and young adults. For example, Bronstein et al. (116) identified 48,177 poison center calls related to caffeine consumption and 6,724 calls related specifically to energy drink consumption. Seifert et al. (115) also reported that 55% of calls regarding caffeine consumption were related to unintentional exposures (115, 116). A study of 13- to 17-year olds admitted to urban emergency rooms in the U.S. found that more than half reported consuming energy drinks in the past month, and those who had were also more likely to report that they had “gotten into trouble at home, school, or work” than those who consumed other types of caffeinated beverages [OR: 3.12 (1.24–7.88)] (117).

In March 2013, 18 scientific and medical experts sent the FDA commissioner a report summarizing the research findings on energy drink consumption in children. This report concluded “… there is neither sufficient evidence of safety nor a consensus of scientific opinion to conclude that the high levels of added caffeine in energy drinks are safe under the conditions of their intended use, as required by the FDA’s Generally Recognized As Safe standards for food additives. To the contrary, the best available scientific evidence demonstrates a robust correlation between the caffeine levels in energy drinks and adverse health and safety consequences, particularly among children, adolescents, and young adults” (118). Furthermore, the Institute of Medicine has recommended that drinks containing caffeine should not be sold to children at school (119). In addition, The American Academy of Pediatrics’ Committee on Nutrition and the Council on Sports Medicine and Fitness recently concluded that “rigorous review and analysis of the literature reveal that caffeine and other stimulant substances contained in energy drinks have no place in the diet of children and adolescents” (120).

### Death

Death from caffeine ingestion appears to be rare. This rarity may be related, in part, to the marked gastric irritation from caffeine that results in spontaneous emesis. Nevertheless, several hospitalizations and some deaths from caffeine toxicity have been reported (121). For example, between 2005 and 2011, there were 79,438 emergency room visits attributable to overconsumption of energy products containing high levels of caffeine in patients aged 12 years and older (121). In most of these cases, the mechanism seems to be tachyarrhythmia and involves unusually high doses of caffeine (>3 mg/kg) (121). Most deaths after caffeine intoxication were caused by overdoses of diet pills and stimulants, and most have occurred in young patients without known underlying heart disease or any variant of normal, such as mitral valve prolapse. In one non-fatal adverse event report, no predisposing factors or structural cardiac abnormality were associated with atrial fibrillation (122). In this case, caffeine-induced atrial fibrillation spontaneously reverted to normal sinus rhythm.

### Reproductive Effects

Caffeine consumption is associated with fertility indices in some studies but not in others. An extensive literature review by the Oak Ridge National Laboratory concluded that chronic caffeine intake in humans is related to adverse effects on conception and reproduction, such as delayed conception and decreased fecundity. These effects appeared at caffeine doses above 200 mg/day (121). A separate review concluded that for healthy adults, intakes below 400 mg/day were not associated with adverse reproductive effects; however, the authors recommended consumption below 300 mg/day for women of reproductive age (123). In addition, some researchers argue that any association between caffeine intake and reproductive outcomes may be explained by other variables, such as maternal smoking or substance use and that research should address confounding, as well as errors in measuring exposure (124).

Reports regarding caffeine consumption and spontaneous abortions have also been conflicting. Weng et al. (125) reported a hazard ratio of 2.23 for spontaneous abortion among 164 women who consumed 200 mg/day or more of caffeine and of 1.34 for 899 women who consumed less than 200 mg/day (125). After adjusting for pregnancy symptoms, such as nausea and vomiting, other researchers found that consuming doses of 200 mg/day or more still almost doubled the risk of spontaneous abortion. A meta-analysis by Chen et al. (126) reported that, compared to a no or very low caffeine intake reference group (0–50 mg/day during pregnancy), every additional 100 mg/day of caffeine (about the amount contained in a typical cup of coffee) increased the risk of pregnancy loss (both miscarriage and stillbirth) by 7% (126). In addition, among women consuming more than 700 mg/day, the risk of pregnancy loss was increased by 72%. Similar findings were reported by Li et al. (127), who found in a separate meta-analysis of 26 studies that the risk of pregnancy loss increased by 19% for every additional 150 mg of caffeine consumed per day and by 8% for every additional 2 cups of coffee (about 200 mg) per day (127). However, Savitz et al. (128) reported no association among 2407 women who were interviewed regarding caffeine intake before experiencing spontaneous abortion (128). This finding suggests that recall bias may explain the increased hazards of spontaneous abortion reported by Weng et al. (125) and potentially other researchers (125). Other comprehensive reviews have reported some evidence that caffeine intakes of more than 300 mg/day have been associated with spontaneous abortion and low birth weight, but all have stressed the need for further research before a causal relationship can be established (129, 130). A recent study from the Nurses Health Study shows pre-pregnancy coffee consumption at levels ≥4 serving/day is associated with an increased risk of spontaneous abortions, particularly at 8–19 weeks gestational age (131).

### Congenital Anomalies

No clear association has been found in humans between moderate doses of caffeine ingestion during pregnancy and birth defects, including congenital heart disease (132). For example, the National Birth Defects Prevention Study found variable results for this possible association (133). In another study of 2,030 malformed infants, the risk of congenital anomalies was not related to the total maternal daily caffeine ingestion below 400 mg/day (or up to 4 cups of coffee) during pregnancy (134). Other studies have found that the frequency of all congenital malformations, including congenital heart defects, was no higher than expected among women who drank between four and eight cups of coffee daily during their pregnancy (135, 136). The Institute of Medicine’s Workshop on Potential Health Hazards Associated with Consumption of Caffeine in Food and Dietary Supplements found that risk of congenital defects from caffeine was not increased in the range of amounts women typically consumed during pregnancy (121).

The consequences of caffeine consumption during pregnancy on offspring have recently been studied in mice (137). Caffeine consumption by the dam (the human equivalent of two to three cups of coffee per day) was associated with caffeine concentrations in the offspring brain that were similar to those in the umbilical cords of women drinking two to three cups of coffee per day (138). At early stages of development, specific types of neurons arise in particular brain regions and then migrate to their target areas. Caffeine slowed the migration of these neurons by 50% by antagonizing adenosine type 2A receptors. As a result, these neurons were late at being incorporated into the circuitry, with negative consequences: pups were more susceptible to seizures, and in adulthood, *in utero* exposed mice had mild cognitive deficits. This study was the first to document that caffeine exposure during pregnancy could harm the offspring. Generalizing the results of animal studies to humans is always speculative, but these results strongly justify conducting prospective studies in humans. Interestingly, in keeping with animal data, greater exposure to caffeine during pregnancy is associated with a lower IQ in children at age 5.5 years (139). This finding again supports the need for additional studies in humans.

### Birth Weight Effects

Several studies have reported a significant negative association between maternal caffeine consumption and birth weight (84, 85, 140–142). However, two other large prospective cohort studies reported a dose-dependent positive association between caffeine intake during pregnancy and the risk of adverse birth weight-related outcomes, such as fetal growth restriction and small for gestational age babies (143, 144). In these studies, caffeine intake and adverse birth weight-related outcomes were found at all amounts of maternal caffeine intake. In both studies, the risk for adverse birth-related outcomes increased notably at a caffeine dose of 200 mg/day from all nutritional sources. In addition, one study of 1,207 pregnant women reported that, although they tended to reduce consumption of caffeine during pregnancy, a moderate decrease in caffeine intake to 100 mg/day in the third trimester of pregnancy did not decrease the risk of adverse birth weight-related outcomes (145).

Two separate meta-analyses of different sets of studies by Rhee et al. (142) and Chen et al. (146) reported odds ratios of having a newborn classified as low-birth weight (less than 2,500 g) for maternal caffeine consumption above 50 mg/day when compared to intakes below 50 mg/day. Furthermore, both meta-analyses found an increased risk of low-birth weight offspring for every 100 mg/day increase in maternal caffeine consumption (OR, 1.03–1.62). Another study by Hoyt et al. (140) found the odds ratios of having a low-birth weight baby increased to a range of 1.3–2.1 in women consuming more than 300 mg/day of caffeine during pregnancy (140).

Taken together, these studies provide substantial evidence of a negative association between maternal caffeine consumption and infant birth weight. Even so, the studies all relied on maternal self-report about caffeine intake; thus, the data may not be accurate. Furthermore, it is possible that additional variables, not controlled for in the analyses, could explain these relationships. For example, chronic sleep loss during pregnancy is also associated with poor birth outcomes, including low birth weight (147). Thus, pregnant women with disrupted sleep might use more caffeine to increase alertness, so the impact on birth weight could be related to short sleep duration and not to caffeine. Although this conclusion is speculative, it highlights the importance of considering additional variables when interpreting correlational data.

### Lactation

Caffeine may cause irritability and sleep disruption in nursing infants whose mothers consume caffeine (148), but the findings are equivocal (149). In addition, some evidence indicates that caffeine intake can reduce production of breast milk (148). Mothers are often advised by their doctors to reduce or eliminate caffeine intake if they feel that their infant shows signs of caffeine sensitivity, but there is no evidence in the literature of detrimental effects of caffeine ingestion during lactation in the general population. Behavioral issues, such as fussiness, jitteriness, and poor sleep patterns, have been reported among infants breastfed by mothers who consumed 10 or more cups of coffee (~1 g of caffeine) per day (121). The effects of caffeine in breast milk can be amplified in preterm infants or infants less than 5 months old because they metabolize caffeine so slowly (121). In addition, an intake of more than 450 mL (about two cups) of coffee per day may decrease breast milk iron concentrations, which could contribute to infant anemia (150). However, the European Food Safety Authority concluded that a single dose of 200 mg or less of caffeine (about two cups) consumed by lactating women, as well as chronic intakes at or below 200 mg, pose no safety concerns for breastfed infants (151).

### Outcomes after Infancy

Few studies have examined the impact of maternal caffeine intake on outcomes after infancy. One study by Klebanoff and Keim (152, 153) using 2,197 mother–child dyads measured child IQ and problem behaviors and examined correlations with maternal paraxanthine concentrations (a metabolite of caffeine) taken between 20 and 26 weeks of gestation (152, 153). This study found no meaningful relationship between maternal caffeine intake during pregnancy and a range of behavioral and cognitive measures in children 4–7 years old. However, another study of 1,083 mother–child pairs revealed that children who were born to mothers who estimated caffeine intake >200 mg/day during pregnancy had an odds ratio of 2.3 (95% confidence interval of 1.13–4.69) of having a child with a lower IQ at age of 5.5 years compared to the reference population of mothers reporting <100 mg/day of caffeine consumption (139). A study by Li et al. (154) reported that maternal caffeine intake was associated with increased odds of childhood obesity, with each 100-mg increase in daily maternal caffeine intake being associated with a 23% higher odds of obesity at age 15 years (127), although a study by Klebanoff and Keim found no relationships between maternal caffeine consumption and childhood obesity (152, 153).

The above studies are correlational; thus, causation cannot be determined. In addition, the maternal caffeine intake in these studies was estimated based on self-reports. One potential explanation for the discrepancies described above is the method used to determine caffeine use. In the study by Klebanoff and Keim (152, 153), which found no significant relationship between maternal caffeine intake and outcomes after infancy, measured serum caffeine concentrations and did not use self-report (152, 153). By contrast, the studies that found significant relationships between maternal intake and measures in the offspring after infancy relied exclusively on retrospective self-reports, several years after the fact, about prenatal caffeine consumption by mothers after they gave birth and during the first two trimesters of pregnancy, respectively. Caffeine intake was estimated from food-frequency questionnaires or interviews in which women reported how often and how much they consumed coffee, tea, and soda. Other variables affecting self-reported caffeine consumption and offspring behavioral outcomes might explain these relationships, but in the study that relied entirely on serum concentrations, such variables were not identified. These studies also measured different outcomes in the offspring. Klebanoff and Keim (152, 153) had the most comprehensive battery of cognitive and behavioral outcomes, but Galera et al. (139) only measured IQ (The Wechsler Preschool and Primary Scale of Intelligence Third Edition), and Li et al. (127) only measured weight and weight gain in the offspring (139, 152–154). Meaningful comparisons of studies are difficult when the methods for assessing caffeine intake and the outcomes are different. Research with objective measures of caffeine intake and standard outcomes is needed.

## Other Existent, Emerging, or Minor Issues

### Cancer

Most of the research examining linkages between caffeine and cancer has been conducted on coffee and tea and not on caffeine specifically, which makes it difficult to determine the mechanism. The International Agency for Research on Cancer has concluded that the evidence is insufficient to conclude that caffeine, as consumed by a typical coffee drinker, is carcinogenic (121). Several large prospective trials have reached the same conclusion (123, 155, 156). Furthermore, Nawrot et al. (123) concluded in their review of the research that caffeine is unlikely to be a human carcinogen at levels less than 500 mg/day, to the equivalent of five cups of coffee (123).

### Unstable Bladder

Excessive caffeine intake (more than 400 mg/day) may increase the risk of detrusor instability (unstable bladder) in women (157). For women with preexisting bladder symptoms, even moderate caffeine intake (200–400 mg/day) may increase the risk for unstable bladder (157). This finding was confirmed in another case–control study of women who were given 200 mg of caffeine citrate (158). In addition, caffeine intake of 4.5 mg/kg/day caused early urgency and frequency of urination in men and women with overactive bladder (159). However, these studies did not examine whether a decrease in caffeine intake was associated with improvements in overactive bladder symptoms. Studies should address this issue.

### Caffeine–Drug Interactions

According to www.drugs.com (a site owned by The Drugsite Trust, a privately held Trust administered by two New Zealand Pharmacists), 85 drugs (430 brand and generic names) are known to interact with caffeine, of which 11 can lead to major interactions.^2^ Because caffeine consumption is at an all-time high and prescription drug use is more prevalent than ever, the risk of negative caffeine and prescription drug interactions is increasing (160, 161). Because of the popularity of caffeine, clinicians should be conscious of the pharmacokinetic interactions between dietary caffeine and over-the-counter and prescription medications, and they should provide the necessary guidance to the patient including dietary restrictions. We also recommend that the potential interaction with these drugs be appropriately addressed on the labeling.

### Hydration and Diuresis

Caffeine has a diuretic effect (123, 162, 163). However, in one clinical trial, different doses of caffeine (up to 6 mg/kg body weight) consumed by 59 habitual caffeine consumers after a 6-day run-in period of 3 mg/kg of caffeine did not markedly change hydration-related biomarkers, suggesting that increasing doses of caffeine did not induce hypohydration in these participants (164). These findings are supported by two similar studies, one in which 5 mg/kg body weight of caffeine was consumed daily for 4 consecutive days by 30 men who normally consumed less than 100 mg/day (42) and one in which 4 mg/kg body weight/day of caffeine from coffee was consumed for 3 consecutive days by 50 adult male habitual coffee consumers who usually consumed 3–6 cups of coffee/day (165). These findings suggest that the diuretic effects from consuming between 4 and 6 mg/kg body weight/day of caffeine are not likely to have adverse consequences for healthy adults who are habitual consumers of caffeine. Similar studies should be conducted in populations that vary by health status, age, and sex.

## Populations At-Risk for Harmful Effects of Caffeine

### Pregnant and Lactating Women

Pregnant women and fetuses may be particularly vulnerable to the effects of caffeine. Caffeine is a biologically active molecule that can act on multiple targets and affect numerous functions positively or negatively. At early stages of fetal development, caffeine may have deleterious effects (137). A recent prospective study suggests that preconception caffeine consumption may also pose a risk to pregnancy, with pre-pregnancy consumption of >400 mg of caffeine/day increasing the risk of spontaneous abortion by 11% compared with women who consumed <50 mg of caffeine/day (131). Many psychoactive compounds can cross the placental barrier and alter the development of the fetal brain. Once caffeine enters the fetal circulation, it is metabolized slowly because neither the placenta nor the fetus itself has cytochrome P450, the enzyme that metabolizes caffeine (166). This reduced caffeine metabolism results in a longer half-life and increased caffeine exposure to the fetus (141, 167). The American College of Obstetricians and Gynecologists recommends limiting caffeine consumption during pregnancy to less than 200 mg/day (168). In the late 1970s, most women maintained their intake during pregnancy at an average of about 190 mg/day^3^ (5). In the 1980s and 1990s, the average maternal caffeine consumption declined to about 125 mg/day (5). Consumption was reported to be about 123 mg/day between 1997 and 2007 (84, 85) and was even lower (58 mg/day) in a 1999 survey (169). This decline has been attributed to FDA warnings that excess caffeine consumption during pregnancy may adversely affect neonates (170). Interestingly, however, in a small cohort of 105 women who drank coffee before pregnancy, 65% reported an aversion to coffee during the first trimester, and 95% voluntarily reduced their consumption during this trimester (171), so perhaps women might be naturally averse to caffeinated products during pregnancy.

Data on caffeine consumption during lactation are limited. One small study from Poland reported that average caffeine intake in a sample of lactating women ranged from 127 to 163 mg/day (172).

### Children and Adolescents

Young children may be vulnerable to the effects of caffeine because they weigh less. For example, a typical can of soda contains about 45 mg of caffeine on average. In an adult weighing 70 kg, the effective dose is 0.6 mg/kg, but in a child weighing 20 kg, the effective dose of the same soda would be 2.25 mg/kg. In comparison, the average caffeine intake in adults is 180 mg/day, resulting in an average effective dose of 2.5 mg/kg. Thus, the physiological impact of a single soda in a child may be equivalent to the impact of two cups of coffee in an average-sized adult. Adolescents may also be particularly vulnerable to the sleep-disrupting effects of caffeine because they may also use caffeinated beverages to stay awake (173, 174).

Data have been collected in children and adolescents using dose–response and placebo-controlled research methods. Outcomes, such as cardiovascular function (175–178), mood (179–181), and cognitive performance (82, 182), have all been measured at caffeine doses ranging from 50 to 300 mg. None of the results suggest that caffeine at these doses is acutely harmful to children and adolescents (183).

Some studies suggest an association between caffeine consumption and longer term behavioral problems in youth, such as anger, violence, sleep disturbances, and alcohol and drug use (180, 184). Researchers in Iceland surveyed 7,400 adolescents (aged 14 and 15 years) and found that most reported consuming caffeine on a typical day and that caffeine intake (primarily from soda and energy drinks) was related to daytime sleepiness and anger for both sexes (185). In a 2013 study of 3,747 15- to 16-year olds, self-reported caffeine intake was strongly associated with self-reported violent behavior and conduct disorders (186). In this study, 21% of participants consumed at least one energy drink per day.

Other studies have found that anxiety can be produced at a wide range of doses (200–2,000 mg of caffeine/day), but many of these studies have used psychiatric patients or patients with a preexisting anxiety disorder (123). Other effects in these studies included nervousness, fidgeting, jitteriness, restlessness, hyperactivity, and sleeplessness (123, 187, 188). When children were stratified by prestudy caffeine intake, emotions and behaviors differed between low- and high-dose consumers (187, 188). Children consuming high doses were more easily frustrated and were more nervous during baseline tests than were the children consuming lower doses. Other studies have found that children with attention-deficit/hyperactivity disorder have higher rates of caffeine abuse, perhaps due to the additive effects of caffeine on dopamine action at the dopamine D2 dopamine receptor, similar to the way guanfacine works for children with this disorder (189, 190).

The safety of high-dose caffeine and energy drinks in younger individuals and caffeine-naïve individuals has not yet been determined. The consumption of highly caffeinated energy drinks has been associated with elevated blood pressure, altered heart rates, and severe cardiac events in children, adolescents, and young adults, especially those with underlying cardiovascular diseases (115, 177, 191, 192). For example, a study of 50 young adults found that consuming a sugar-free energy drink containing 80 mg of caffeine (slightly less than the caffeine contained in one cup of coffee) was associated with changes in platelet and endothelial function great enough to increase the risk for severe cardiac events in susceptible individuals (193). These findings show how the acute effects of caffeine on heart rate might result in cardiovascular events requiring hospitalization, especially in at-risk young adults. In addition, caffeine’s effects on blood pressure are more pronounced among African-American children than among Caucasian children (mean difference in blood pressure averaging 6.5 mm Hg) (175, 194). High doses of caffeine may exacerbate cardiac conditions for which stimulants are contraindicated (195–198). In particular, ion channelopathies and hypertrophic cardiomyopathy, which is the most prevalent genetic cardiomyopathy in children and young adults (0.2% of the population), are of concern because of the risk of hypertension, syncope, arrhythmias, and sudden death (197, 199).

### Patients with Mental Illness

Another population that may be at risk for adverse effects of caffeine are patients with mental illness. Caffeine antagonism of adenosine receptors can result in enhanced dopaminergic signaling, thought to be due to a combination of increased dopamine release (200, 201), upregulation of dopamine receptors, and increased affinity of dopamine receptors for dopamine in the striatum and nucleus accumbens (202). Furthermore, adenosine receptors can form heterodimers with dopamine receptors (203), which can modulate dopamine signaling. For some psychiatric illness, such as Parkinson’s disease, Alzheimer’s disease, and depression, caffeine antagonism of adenosine receptors may improve symptoms (204, 205) and slow the progression of neurodegeneration (206, 207), although these findings are equivocal with some studies reporting caffeine increases depressive symptoms (208). For other mental illness, such as schizophrenia, caffeine may exacerbate psychotic symptoms (209), although the majority of this literature is informed by case studies, with very few double-blind placebo-controlled studies (210). There is also good evidence that higher caffeine use is associated with greater reporting of anxiety symptoms (211, 212) and may increase risk of symptom relapse (213) and suicide among bipolar disorder patients (214). Finally, there is strong empirical evidence that caffeine potentiates the rewarding effects of drugs of abuse (215–217), which suggests that caffeine use can increase vulnerability to substance use disorder (218). The lack of randomized control trials on the impact of caffeine in patients with mental illness makes it difficult to determine safe doses, effects of acute and chronic caffeine, and potential interactions between caffeine and medications. Currently, there are no specific recommendations for caffeine consumption for individuals with mental or psychiatric illness, but it may be worth consideration by physicians and psychologists treating patients with mental illness.

## Caffeine and Alcohol

Another increasingly popular form of caffeine consumption is to mix alcohol with energy drinks. In fact, there are several recent reviews on this topic (219–221). We will briefly highlight this literature here. In 2010, the FDA removed pre-mixed alcohol-energy drinks from the market because caffeine was determined to be an unsafe additive to alcohol,^4^ in part because it promoted excessive drinking (222). However, energy drinks can be legally mixed with alcohol in the U.S. if they are sold separately. In fact, this practice is popular among college students, as suggested by the increase in self-reports over the past 5–10 years (223–229). The research on alcohol-mixed energy drinks is still developing, and the vast majority has been conducted in the U.S. and Australia. Much of this research consists of surveys of college-age young adults immediately after they leave bars where they have been drinking (230–233). Self-report is often unreliable, but self-report while intoxicated may be particularly problematic. Similarly, intoxication may confound retrospective assessments of alcohol consumption and related behaviors and attitudes.

More recently, several well-controlled, objective, laboratory-based studies on the impact of alcohol-mixed energy drinks have been conducted. In many studies, the combination of alcohol and energy drinks results in higher rates of binge drinking, reductions in perceived intoxication, faster rates of self-paced alcohol consumption, or increases in risk taking behavior (225, 234–239). These data are equivocal, however, with studies showing that caffeine combined with alcohol does not always increase the amount of alcohol consumed (240) or does not have an impact on risk taking behavior (235, 241). Potential reasons for these discrepancies may be difference in the doses of caffeine and alcohol, differences in the administration paradigm, and an influence of expectancy of caffeine effects on alcohol intoxication (241). More work is needed in this area to be able to draw stronger conclusions.

## Caffeine-Related Diagnoses

The American Psychiatric Association’s Diagnostic and Statistical Manual-IV (242) included four caffeine-related diagnoses: caffeine intoxication, caffeine-induced anxiety disorder, caffeine-induced sleep disorder, and caffeine-related disorder not otherwise specified (242). Caffeine intoxication is diagnosed if clinically significant impairment results from the following criteria: (1) recent consumption of caffeine, usually in excess of 250 mg, (2) five (or more) of the following: restlessness, nervousness, excitement, insomnia, flushed face, diuresis, gastrointestinal disturbance, muscle twitching, rambling flow of thought and speech, tachycardia or cardiac arrhythmia, periods of inexhaustibility, psychomotor agitation, and (3) the symptoms in criteria (2) have to cause clinically significant distress or impairment in social, occupational, or other important areas of functioning and these symptoms cannot be attributable to another medical condition or mental disorder. Caffeine-induced anxiety and sleep disorder retain the diagnosis for substance/medication-induced anxiety and sleep disorders, but require that clinically significant symptoms occur in association with caffeine intoxication or withdrawal (243). Caffeine-related disorder not otherwise specified classifies symptoms related to caffeine use or withdrawal that do not fit into the aforementioned categories.

The latest edition of the DSM (243) has officially recognized caffeine withdrawal disorder and outlines guidelines for criteria for caffeine use disorder in a section on emerging measures and models (243). The diagnosis of caffeine withdrawal syndrome is empirically based on detailed analyses of decades of studies of symptoms [reviewed by Juliano and Griffiths (244)]. Caffeine withdrawal disorder is diagnosed when an individual experiences clinically significant impairment related to withdrawal symptoms after abrupt cessation of caffeine intake, including headache, difficulty concentrating, fatigue, nausea, flu-like symptoms, and changes in mood. These symptoms typically begin 12–24 h after caffeine cessation and may continue for 3–7 days. Ongoing research on caffeine withdrawal suggests that this continues to be an important problem and will help refine and clarify this diagnosis (245, 246). Avoidance of caffeine withdrawal, with or without a diagnosis of caffeine withdrawal disorder, may motivate individuals to consume more caffeine. This could result in chronic, excessive consumption of caffeine. When this excess consumption results in clinically significant impairment, an individual may meet the criteria for caffeine use disorder (247–249). Although not an official DSM diagnosis, the proposed criteria for caffeine use disorder include having all three of the following criteria met: (1) persistent desire or unsuccessful effort to control caffeine use, (2) “use despite harm,” and (3) withdrawal. Having these proposed criteria outlined will allow researchers to collect data to provide reliable and valid empirical studies of the prevalence of this phenomenon (250). This is critical because the progression of inclusion of caffeine-related diagnoses is directly related to an increase in empirical support for such disorders.

## Recommendations on Safe Intake Levels and Limits on Intake

Caffeine reaches maximum plasma concentration 15–120 min after ingestion (251), which might explain why energy drink-related adverse events are usually reported a few hours after consumption. The threshold of caffeine toxicity appears to be around 400 mg/day in healthy adults (19 years or older), 100 mg/day in healthy adolescents (12–18 years old), and 2.5 mg/kg/day in healthy children (less than 12 years old) (123, 192). For comparison, one standard sized can of a popular energy drink provides 77 mg of caffeine (or 1.1 mg/kg/day) for a 70-kg male and twice that, 2.2 mg/kg/day, for a 35-kg pre-teen (252). Recommended safety thresholds vary, however. For example, the European Food and Safety Authority considers 3-mg/kg body weight/day of habitual caffeine consumption to be safe for children and adolescents (253).^5^

A comprehensive review of the effects of caffeine consumption on human health concluded that for healthy adults, moderate chronic intakes of caffeine up to 400 mg/day are not associated with adverse effects on cardiovascular health, calcium balance and bone status, behavior, cancer risk, or male fertility (123). However, the recommended intake is much lower for pregnant or nursing mothers. The European Commission’s Scientific Committee of Food Safety Authority and Health Canada both recommend that women consume no more than 300 mg of caffeine/day during pregnancy (121, 253). In addition, despite conflicting results regarding the association between caffeine consumption and spontaneous abortion, the American College of Obstetricians and Gynecologists recommends that pregnant women restrict their caffeine intake to less than 200 mg/day (121).

For most children, adolescents, and young adults, safe levels of caffeine consumption have not been established. Because deleterious effects of heavy caffeine use have been documented in those who have cardiovascular issues, studies of safe doses and the effects of chronic use are paramount in understanding the implications of caffeine. This research should seek to better characterize the effects of caffeine use before, during, and after exercise, the interactions of caffeine use with alcohol and medications, such as stimulants, and the effects of prolonged caffeine use. A better understanding of caffeine’s effects in individuals with cardiac problems will better equip health-care providers to screen and identify at-risk individuals, and in turn, to better educate and counsel these cardiac patients. Such information will also help health-care leaders to work with families, schools, and other community services to change marketing strategies, improve the dissemination of information, and identify at-risk behaviors and age groups. Finally, the health-care providers and regulatory agencies must begin collecting and archiving better data on the adverse events and health effects of caffeine consumption to improve estimates about its scope, effects, and outcomes. Analyses of a comprehensive, centralized database would help direct research, education, and funding to support these populations. In addition, agencies like the U.S. FDA and Health Canada need to initiate programs to educate consumers, especially children and adolescents, about the dangers of highly caffeinated products, to reconsider applying the U.S. FDA’s Generally Recognized as Safe standard to energy drinks and other beverages with added caffeine, and requiring manufacturers to include the caffeine content on product labels. Because of the potentially harmful adverse effects and developmental effects of caffeine, the consensus among the research and medical communities is that any dietary intake of caffeinated energy drinks should be discouraged for all children (123, 192).

One of the primary concerns about energy drinks is that the actual caffeine content is not often given on the product’s packaging or on its website (120). The total amount of caffeine contained in some energy drinks can exceed 500 mg (equivalent to 14 cans of common caffeinated soft drinks or 5 cups of coffee) and is high enough to be toxic in children and young adults (34). Given these concerns, the American Academy of Pediatrics released the following recommendation to the United States Senate Committee on Commerce, Science, and Transportation:

Due to the potentially harmful health effects of caffeine, dietary intake should be discouraged for all children. Because the actual stimulant content of energy drinks is hard to determine, energy drinks pose an even greater health risk than simple caffeine. Therefore, energy drinks are not appropriate for children and adolescents and should never be consumed (2014).

In 2010, Health Canada convened an Expert Panel^6^ on Caffeinated Energy Drinks to develop a plan to more effectively address the safety concerns related to caffeinated energy drinks currently marketed in Canada. The Panel issued their recommendations to Health Canada in the fall of 2010.^7^ Health Canada analyzed the recommendations, completed a health risk assessment, and continued to gather and exchange information with major food safety regulators within the country and internationally. This initiative resulted in a proposed management approach that was consistent with the strategies in the Panel’s recommendations. Components of this approach include regulating product formulation and labeling, addressing potential health risks and adverse effects, providing enhanced education and communication to consumers, and addressing uncertainties and data gaps through research on long-term effects. Long-term research was made a priority, to further investigate risks to consumers, to identify serious adverse event signals (such as cardiac events and to a lesser extent, seizures), and finally to better manage caffeine labeling and dosing limits. The data have reconfirmed that moderate daily caffeine intake at dosages of up to 400 mg/day are not associated with adverse effects. However, the data show that women of childbearing age and children may be at higher risk from caffeine, which has therefore led to separate guidelines for these at-risk groups. However, several products containing stimulant drugs do not have a natural health product license and exemption numbers that clearly describe their caffeine content. Therefore, the Panel recommended that Health Canada ensure that all products meet strict labeling that includes a full disclosure of the exact caffeine dose. Finally, the Panel recommended that Health Canada, in collaboration with the provinces and territories, consider beginning a surveillance system in sentinel emergency rooms across the country to actively search for serious adverse drug reactions associated with consuming drinks containing stimulant drugs with or without alcohol or other products. The proposal details how this system could be modeled after the nation’s long-running IMPACT system that monitors immunizations and related adverse events through a network of 12 Canadian centers, representing 90% of all tertiary care pediatric beds. A similar database, The Canadian Health Measures Survey,^8^ launched in 2007, contains data from voluntary household interviews that collects important health information (e.g., physical measurements, nutrition, and blood and urine samples).

## Future Research

Several questions remain about caffeine consumption and patterns of intake. First, it is not clear how much caffeine is being consumed from “uncommon” or unidentified sources of caffeine, such as foods and medications. These sources are often overlooked in large national surveys and, thus, caffeine intake may be underestimated. Second, caffeine may be indirectly harmful because it is consumed with other substances that are harmful. For example, coffee drinking may promote donut eating or cigarette smoking, or energy drink consumption may promote alcohol intake. Third, future studies need to investigate absorption, distribution, metabolism, and excretion of caffeine occurring in non-natural forms (such as encapsulated forms), which may influence pharmacokinetics, and thus effects. Finally, most research has relied on self-report and correlational analysis, which limits the ability to determine causality and directionality.

Despite all that is known about caffeine intake and safety of caffeine consumption, certain gaps in our knowledge need to be addressed:

(1) *Identifying at-risk populations for caffeine toxicity*. We already know that small children and pregnant women, as well as individuals with cardiac or vascular disease, are likely to be particularly vulnerable to the harmful effects of caffeine. Furthermore, there is some evidence that individuals with mental illness may also be at risk for harmful effects of caffeine on symptoms, but the majority of these relationships have been described in case studies. More randomized control trials need to be conducted in patients with mental illness to determine safe doses for caffeine ingestion. In addition to the known vulnerable populations, there may be individuals, such as the elderly or individuals with underlying medical conditions, who are not part of any vulnerable population but who, for genetic or metabolic reasons, may be susceptible to harmful effects. The Federal Substance Abuse and Mental Health Services Administration reported that from 2007 to 2011, the number of emergency room visits involving energy drinks doubled across the U.S., from 10,068 to 20,783. However, for adults aged 40 years and older, emergency room visits involving energy drinks nearly quadrupled during that same period (from 1,382 to 5,233).^9^ This finding suggests that energy drink consumption in older people is increasing with perhaps a greater risk of negative outcomes. Identifying and warning at-risk individuals to avoid caffeine-containing products would be desirable.
(2) *Determining how best to disseminate information about caffeine content in a meaningful and truthful way without causing alarm*. Although the preponderance of evidence suggests that caffeine is safe for most people, there may be reasons to limit caffeine use in some populations. Providing more information about safe levels may be useful, but the information must be understandable to the population and based on evidence, rather than on supposition. Adding information about caffeine content on the products themselves may not be enough. The best way to educate consumers about safe levels of caffeine consumption needs to be determined. For example, evidence suggests that “natural frequencies” are an effective way to communicate risk. For example, one could explain “For every 1,000 children who consume energy drinks, XX will have CNS symptoms.” However, research is necessary to fill in the blank in this statement (254).
(3) *Conducting prospective, longitudinal studies to determine how caffeine use relates to behavioral and health-related outcomes*, such as the duration and quality of sleep, potential for abuse, and impact on the use of other substances, including controlled (cigarettes and e-cigarettes) and uncontrolled (marijuana, cocaine) drugs. Cross-sectional data suggest that caffeine use is generally safe, but rigorous longitudinal studies have not yet determined the effect of chronic caffeine consumption on development in children and adolescents.
(4) *Further exploring the potential health benefits of caffeine*. Although much of this document has focused on potential harmful effects of caffeine, some health benefits of caffeine remain under explored. In particular, some research suggests that caffeine may slow age-related cognitive decline (255, 256), reduce risk of some neurological disorders (90, 257, 258), and promote longevity (156).
(5) *Developing better systems of documenting and sharing adverse events*. In addition to identifying at-risk or vulnerable populations, as mentioned earlier, and potentially dangerous combinations of caffeine with other substances (e.g., alcohol), we need a better system of documenting adverse events and sharing that documentation among scientists and clinicians. Systematically collecting all adverse events, poison center data, and emergency room visits associated with caffeine consumption (for example, energy drink consumption), together with more comprehensive evaluation of additional risk factors, is necessary to accurately determine the risks of toxicity for youth and other vulnerable individuals.
(6) *Improving knowledge of the potential dangers from consuming energy drinks before, during, and after athletic activity* will be essential to identify the potential dangers of direct and implied claims of enhanced athletic performance, which is common in energy drink marketing. Long-term systematic assessment of energy drink and general caffeine intake at the population level, specifically intake by youth, should be a priority.

## Conclusion

When taken together, the literature reviewed here suggests that ingested caffeine is relatively safe at doses typically found in commercially available foods and beverages. There are some trends in caffeine consumption, such as alcohol-mixed energy drinks, that may increase risk of harm. There are also some populations, such as pregnant women, children, and individuals with mental illness, who may also be considered vulnerable for harmful effects of caffeine. Excess caffeine consumption is increasingly being recognized by health-care professionals and by regulatory agencies as potentially harmful. More research needs to be conducted to address these emerging concerns and provide empirical support for the recommendations.

## Author Contributions

JT, CB, and SL contributed equally to the preparation of this comprehensive review. JC, JW, and MM helped gather additional references and prepare the manuscript after the initial major review of the literature was conducted.

## Conflict of Interest Statement

The authors prepared this comprehensive review at the request of the American Association for the Advancement of Science. Once the draft was completed, we were given permission to publish the manuscript. SL has served as an expert for legal cases involving caffeine-containing energy drinks.

## Footnotes

^1^https://www.fda.gov/ForConsumers/ConsumerUpdates/ucm350570.htm.

^2^https://www.drugbank.ca/drugs/DB00201#pharmacology.

^3^It is difficult to calculate the caffeine intake relative to body weight during pregnancy because women begin pregnancy at a broad range of weights, gain weight at different rates, and gain different amounts of weight. Because of this, only absolute caffeine intake is shown in this section.

^4^https://www.fda.gov/NewsEvents/PublicHealthFocus/ucm234900.htm.

^5^http://www.efsa.europa.eu/en/efsajournal/pub/4102.

^6^http://www.hc-sc.gc.ca/dhp-mps/prodnatur/activit/groupe-expert-panel/index-eng.php.

^7^http://www.hc-sc.gc.ca/fn-an/securit/addit/caf/ced-response-bec-eng.php.

^8^http://www23.statcan.gc.ca/imdb/p2SV.pl?Function=getSurvey&SDDS=5071.

^9^http://www.samhsa.gov/data/sites/default/files/DAWN126/DAWN126/sr126-energy-drinks-use.pdf.
